# Functionalization of Photopolymer with Laser-Ablated Copper NPs: A Comprehensive Study of ROS Generation, Antimicrobial Activity and Cytotoxic Profile

**DOI:** 10.3390/polym18020238

**Published:** 2026-01-16

**Authors:** Dmitriy E. Burmistrov, Dmitriy A. Serov, Lev R. Sizov, Maxim E. Astashev, Ekaterina E. Karmanova, Ilya V. Baimler, Alexander V. Simakin, Dmitriy N. Ignatenko, Fatikh M. Yanbaev, Evgeny V. Kuzmin, Sergey V. Gudkov

**Affiliations:** 1 Prokhorov General Physics Institute of the Russian Academy of Sciences, Vavilov Str. 38, Moscow 119991, Russia; dmitriiburmistroff@gmail.com (D.E.B.); dmitriy_serov_91@mail.ru (D.A.S.); leo.sizoff@yandex.ru (L.R.S.); astashev@yandex.ru (M.E.A.); ilyabaymler@yandex.ru (I.V.B.); avsimakin@gmail.com (A.V.S.); dmitriyek13104@yandex.ru (D.N.I.); 2 Federal Research Center Pushchino Scientific Center for Biological Research, Institute of Cell Biophysics, Russian Academy of Sciences, 3 Institutskaya St., Pushchino 142290, Russia; silisti@bk.ru; 3 Federal Research Center Kazan Scientific Center, Russian Academy of Sciences, ul. Lobachevskogo 2/31, Kazan 420088, Russia; aurum_fr@mail.ru; 4 Lebedev Physical Institute, Russian Academy of Sciences, 58 Leninskiy Av., Moscow 119991, Russia; 5 Department of Fundamental Sciences, Bauman Moscow State Technical University, 5 2nd Baumanskaya St., Moscow 105005, Russia

**Keywords:** zero-valent copper, bioactive polymers, functional material, stereolithography, antibacterial surfaces

## Abstract

This study addresses the critical need for advanced biomedical materials that possess both potent antimicrobial properties and high biocompatibility to prevent device-related infections and promote healing. To this end, we demonstrate the successful development and comprehensive characterization of functional composite materials based on a photopolymerizable acrylate resin modified with laser-ablated copper nanoparticles (Cu NPs). The synthesized Cu NPs exhibited a monomodal size distribution with a peak at 47 nm, a high zeta potential of −33 mV, and a spherical morphology. Incorporation of Cu NPs into the polymer matrix via Masked Stereolithography (MSLA) enabled the fabrication of complex structures that maintained high surface quality and optical transparency after polishing. Modification of photopolymer resin with Cu NPs significantly increased the strength of the resulting products and caused dose-dependent formation of reactive oxygen species (ROS). The resulting composite materials exhibited strong antibacterial activity against *E. coli*. Crucially, despite their potent antimicrobial efficacy, the materials showed no cytotoxicity towards human fibroblast cultures. These results highlight the potential of these composites for a new generation of biomedical applications, such as implantable devices and wound coatings, which combine programmable antimicrobial activity with high biocompatibility.

## 1. Introduction

The contemporary advancement of additive manufacturing technologies is stimulating an active search for novel multifunctional materials that combine enhanced physicochemical properties with targeted biological activity [1,2]. In the context of the rapid progress in photopolymer printing technologies, research focused on the modification of methacrylate-based photopolymerizable resins is of particular significance. These resins represent promising polymer systems that have found widespread application in medical practice due to their favorable combination of biocompatibility and processability [3,4]. One of the most effective current strategies for improving such materials is the incorporation of nanoscale additives capable of purposefully altering the functional characteristics of the polymer matrix [5,6,7].

Among the diverse types of nanomaterials, copper nanoparticles (Cu NPs) deserve special attention, as they demonstrate pronounced antimicrobial activity against a broad spectrum of pathogenic microorganisms, including Gram-positive and Gram-negative bacteria, as well as pathogenic fungi [8,9]. From a biomedical perspective, the features of Cu NPs include their high bioavailability and moderate toxicity [10,11]. Copper possesses a range of unique physical properties, among which its low susceptibility to corrosion, exceptional thermal and electrical conductivity, and high ductility are particularly notable [12,13]. Ionic forms of copper exhibit significant reactivity, forming stable compounds with various organic substances, which can disrupt natural ecological and physiological processes [14].

Copper ions in the +1 and +2 oxidation states (Cu^+^ and Cu^2+^) are of particular biological interest, as they are integral components of the active sites of numerous enzymes and proteins [15,16]. These ions perform a critical function in living systems by serving as electron carriers in the mitochondrial respiratory chain [17]. Copper ions play a fundamental role in key metabolic pathways, including protein biosynthesis, regulation of cholesterol metabolism, and the metabolism of iron and carbohydrates [18]. Copper is especially important in hematopoiesis, where it participates in hemoglobin synthesis, as well as in the formation and maintenance of connective tissue integrity [19]. It is important to note that, unlike most microorganisms, mammals possess the serum protein ceruloplasmin, which normally serves as the most extensive depot for copper ions in the body, containing 90 to 95% of serum copper [20].

Metallic copper in the zero-valent state (Cu^0^) constitutes an ordered crystalline structure formed by a multitude of reduced copper ions. The most stable form of such a system is spheroidal nanoparticles, which demonstrate high structural integrity. Metallic copper nanomaterials have found widespread practical application as functional additives for lubricants [21] and polymer composites [22,23]. The antimicrobial activity of copper nanoparticles (Cu NPs) is directly related to their nanoscale characteristics and their ability to release ions in a controlled manner under acidic conditions [24]. A specific mechanism of the antimicrobial action of Cu NPs is attributed to their ability to generate reactive oxygen species and disrupt microbial cell membranes [25,26]. Furthermore, Cu NPs exhibit a pronounced oligodynamic effect—the ability to exert a bactericidal action at minimal concentrations [27,28]. In addition to their biological activity, Cu NPs possess unique physicochemical properties, including high electrical conductivity and catalytic activity, which expand their potential for practical applications [29,30].

The incorporation of Cu NPs into photopolymerizing composites opens avenues for creating materials with programmable surface properties that confer resistance to bacterial adhesion and biofilm formation. However, the widespread adoption of such technologies in clinical practice is hampered by a limited selection of materials that simultaneously meet the demanding technological requirements of printing, possess the necessary mechanical properties, and, crucially, exhibit targeted biological activity to prevent infectious complications. This study addresses this complex challenge. Its key motivation is to create bioactive photopolymer composites for additive manufacturing that combine the processability of 3D printing with pronounced antimicrobial properties, without compromising cytocompatibility. Modification of promising methacrylate resins with Cu NPs was chosen as a strategic approach to achieving this goal. This choice is justified by the fact that, despite the known antimicrobial potential of copper, systematic studies devoted to the integration of Cu NPs into photopolymerizable 3D printing systems remain fragmented. There is a lack of comprehensive data on the influence of nanoscale copper additives on the photopolymerization process, the final physicochemical properties of printed objects, and the balance between antimicrobial efficacy and biocompatibility of the resulting material.

In this regard, the present study is aimed at the additive manufacturing of products from modified photopolymerizable resins based on acrylic oligomers by incorporating Cu NPs at various concentrations, as well as a comprehensive investigation of the structure–property–bioactivity relationships in the obtained materials. The work involves the synthesis and characterization of nanoparticles, the development of a methodology for their incorporation into the photopolymer resin, the additive manufacturing of prototype specimens, and a comprehensive study of the physicochemical properties, antimicrobial activity, and cytocompatibility of the developed materials.

## 2. Materials and Methods

### 2.1. Synthesis of Copper Nanoparticles (Cu NPs) Colloid and Incorporation into Photopolymer Resin

Cu NPs were synthesized by laser ablation of a high-purity copper target (99.99%) in acetone. Ablation was performed using a fiber ytterbium laser with the following parameters: λ = 1064 nm, f = 35 kHz, τ = 200 ns, Ep = 1.5 mJ, and a laser spot size at the beam waist of 50 μm. The typical irradiation time for the targets was 30 min. During irradiation, the laser beam was scanned across the target surface using a galvanometric scanner (LScanH, Ateko-TM, Moscow, Russia) and an F-theta lens. The beam trajectory consisted of several parallel lines inscribed within a square, with a hatching resolution of 70 lines/mm and a scanning speed of 3000 mm/s.

The synthesized nanoparticles were characterized using a suite of analytical techniques. Size distribution, zeta potential, and particle concentration were determined using a Malvern Zetasizer Ultra analyzer. The optical properties of the colloidal solution were studied by UV-Vis spectroscopy on a CINTRA 4040 (GBC Scientific Equipment Pty Ltd., Keysborough, Australia) instrument. The morphological features of the nanoparticles were investigated using a Libra 200 FE HR transmission electron microscope (Carl Zeiss, Jena, Germany). Certain aspects of the nanoparticles synthesis and characterization have been described previously [31].

The resulting nanoparticles dispersion in acetone was mixed with a ClearPro photopolymerizable acrylate resin (Harz Labs, Mytishchi, Russia) to achieve target concentrations of 0.001%, 0.01%, and 0.1% by weight. The base resin was a transparent liquid with a viscosity of 1000 ± 200 mPa·s. According to the manufacturer’s data, the commercial resin used had the following composition: 50–70% oligourethane (meth)acrylate, 30–50% polyethyleneglycol dimethacrylate, 1–5% 2-hydroxypropyl methacrylate, and 1–3% photoinitiator. The exact names of the components, their CAS numbers, and EC numbers were not provided, as they are trade secrets of the manufacturer.

To ensure uniform distribution of nanoparticles within the polymer matrix, the mixtures were subjected to mechanical stirring on a laboratory shaker followed by ultrasonic treatment at 40 kHz and 22 °C for 5 min. The prepared modified resins were stored in sealed, dark glass vials protected from light.

### 2.2. Additive Manufacturing of Specimens from Modified Composite Materials

Specimens were fabricated from the modified photopolymer resins using Masked Stereolithography (MSLA) technology on a Saturn 3 Ultra 12 K printer (Elegoo, Shenzhen, China). Prior to the printing cycle, the prepared modified resin with specified nanoparticles concentrations was poured into the printer’s vat. For comparative analysis, a control group of specimens was simultaneously manufactured from the unmodified base resin without nanoscale additives. Two types of specimens were printed: (1) round plates with a diameter of 16 mm and a thickness of 0.5 mm, intended for comprehensive physicochemical and biological studies, and (2) 3D structures with complex lattice architecture to determine the maximum achievable printing resolution using the composite resin.

The printed objects were washed in absolute isopropanol using a magnetic stirrer for six minutes. This was followed by ultrasonic cleaning in absolute isopropanol for the same duration, after which the specimens were air-dried at room temperature for ten minutes. The post-processing stage involved the application of glycerol, followed by UV post-curing on a rotating platform for thirty minutes. Subsequently, a second ultrasonic cleaning and drying step was performed. The final step was heat treatment in an oven at 80 °C for thirty minutes. The finished specimens, ready for testing, were placed in covered Petri dishes and stored under standard laboratory conditions to preserve their properties throughout the study period. Certain specifics of specimen fabrication and post-processing have been described previously [32].

### 2.3. Physicochemical Characterization of Specimens Printed from Modified Resins

The micro- and nanostructure of the surface of specimens obtained from composite materials with Cu NPs additives was investigated using an NT-MDT atomic force microscope (AFM) analytical complex (NT-MDT LLC, Zelenograd, Russia) in semi-contact scanning mode. The distribution of Cu NPs within the polymer matrix volume was analyzed using a MIM-321 modulation-interference microscope (Amphora laboratories, Moscow, Russia), providing detailed information on the presence of hidden defects and the nature of nanoscale additive dispersion within the polymer. Some experimental details have been described previously [33]. Uniaxial tensile mechanical tests were conducted on a WDW-5S universal testing machine (Hongtuo, Dongguan, China). Specimen preparation and testing procedures were strictly followed according to ASTM D638-22. Tests were conducted at room temperature with a constant crosshead speed of 2 mm/min. To ensure statistical reliability of the results, at least five identical specimens were tested for each material composition. Load–displacement data were recorded and subsequently processed to obtain mechanical properties using specialized software included with the testing equipment. Raman spectra were recorded on a Senterra II Raman microscope (Bruker, Billerica, MA, USA) with an excitation wavelength of 785 nm, a 20× objective (NA = 0.4), and a spectral resolution of 1.5 or 4.0 cm^−1^.

### 2.4. Quantitative Assessment of Reactive Oxygen Species (ROS) Generation in Aqueous Solutions

The generation of reactive oxygen species, specifically hydrogen peroxide (H_2_O_2_) and hydroxyl radicals (^•^OH), in aqueous solutions after contact with Cu NPs-based composite materials was investigated using highly sensitive analytical methods. Hydrogen peroxide concentration was determined by chemiluminescence using a Biotox-7A-USE (ANO “Engineering Center—Ecology,” Moscow, Russia) instrument. Measurements were performed in a reaction system containing luminol, 4-iodophenol, and horseradish peroxidase in Tris-HCl buffer at pH 8.5 [34]. Printed specimens were placed in polypropylene vials with distilled water and incubated at 40 °C for three hours. A freshly prepared counting solution containing Tris-HCl buffer, para-iodophenol, luminol, and horseradish peroxidase was then introduced into the system. This method is exceptionally sensitive, capable of detecting trace amounts of hydrogen peroxide below 0.1 nM.

For the quantitative determination of hydroxyl radicals, a fluorimetric method was employed using coumarin-3-carboxylic acid as a fluorescent probe [34]. Thin film samples were incubated with the probe solution in phosphate-buffered saline at 80.0 ± 0.1 °C for two hours. The resulting fluorescent product, 7-hydroxycoumarin-3-carboxylic acid (CCA), was detected using a JASCO 8300 (JASCO, Tokyo, Japan) spectrofluorometer. Control measurements without samples were performed in all experiments to account for background signals. A series of composite materials with Cu NPs concentrations ranging from 0.001 to 0.1 wt.% was studied, with each analysis performed in triplicate to ensure statistical reliability.

### 2.5. Quantitative Determination of 8-Oxoguanine in DNA

The quantitative determination of 8-oxoguanine content in DNA samples was performed by enzyme-linked immunosorbent assay (ELISA) using specific monoclonal antibodies [35]. Sample preparation began by adjusting DNA samples to a concentration of 350 µg/mL, followed by denaturation by heating in a water bath for 5 min and rapid cooling on ice. For analysis, 42 µL of the prepared DNA was added to the wells of a microplate, where the molecules were immobilized at 80 °C for 3 h. Non-specific binding sites were blocked with a 1% solution of skimmed milk in Tris-HCl buffer (pH 8.7) containing 0.15 M NaCl for 14–18 h at room temperature. In the next step, primary antibodies against 8-oxoguanine, diluted 1:2000, were added to the wells and incubated at 37 °C for 3 h. After washing, secondary antibodies conjugated with horseradish peroxidase (dilution 1:1000) were added, followed by incubation at 37 °C for 1.5 h. The enzymatic reaction was detected using the substrate ABTS (18.2 mM) in the presence of 2.6 mM H_2_O_2_ in 75 mM citrate buffer (pH 4.2). The reaction was stopped by adding 100 µL of 1.5 mM sodium azide upon color development. Optical density was measured at a wavelength of 405 nm using a Feyond-A400 microplate reader (Allsheng, Hangzhou, China).

### 2.6. Detection of Long-Lived Reactive Protein Species (LRPS)

The assessment of changes in the concentration of long-lived reactive protein species was carried out using a chemiluminescence method [36]. This method is effective and sensitive for determining free radical reactions. A Biotox-7A-USE chemiluminometer (ANO “Engineering Center—Ecology”, Moscow, Russia) was used to study long-lived reactive protein species by measuring the chemiluminescence of bovine serum albumin solutions upon temperature increase. Chemiluminescence measurements were performed in the dark at room temperature in 20 mL plastic polypropylene vials (Beckman, Brea, CA, USA). The test material samples were pre-heated in a water bath to 45 °C for 2 h. Non-heated protein solutions were used as controls.

### 2.7. Microbiological Studies of Antibacterial Activity

The antibacterial properties of material samples with and without Cu NPs were evaluated using a test system based on 24-well plates [33]. Preliminary sample preparation included treatment with 70% ethanol followed by UV sterilization in a laminar flow hood for 40 min. *Escherichia coli* bacterial culture was used for the studies. An overnight bacterial culture was grown in sterile LB broth with constant shaking at 230 rpm and a temperature of 37 °C. Immediately before the experiment, the obtained culture was diluted with fresh nutrient medium to a concentration of approximately 10^6^ CFU/mL. A volume of 1000 µL of the bacterial suspension was added to each well containing the test material sample. The uncovered plate was placed in a Feyond-A400 (Allsheng, Hangzhou, China) plate photometer equipped with a thermostating and shaking system. Over a 24 h incubation period at a constant temperature of 37 °C, the optical density of the bacterial culture was automatically measured at a wavelength of 600 nm. Based on the obtained data, growth curves were plotted and analyzed, with particular attention to parameters such as lag phase duration, exponential growth rate, and maximum bacterial culture density. Control groups included pure polymer samples without nanoparticles and sterile nutrient broth.

Additional studies were conducted using flow cytometry. After the main cultivation stage, a phosphate-buffered saline containing 4 µM of the fluorescent dye propidium iodide (Lumiprobe, Westminster, MD, USA) was added to each sample. The samples were incubated in the dark for 60 min, then resuspended and analyzed on a Longcyte CLQC-281 flow cytometer (Challenbio, Beijing, China). The used dye is characterized by a significant increase in quantum yield upon binding to DNA, allowing for accurate assessment of bacterial cell viability after contact with the test materials.

### 2.8. In Vitro Cytotoxicity Assessment of Composite Materials

The cytotoxic properties of material samples with and without Cu NPs were studied using a culture of Human Spleen Fibroblasts (HSF). Cell lines were cultured under standard conditions using DMEM/F12 nutrient medium supplemented with fetal bovine serum, L-glutamine, and antibiotics (PanEco, Moscow, Russia). Prior to the experiment, laboratory glassware was carefully prepared: round cover glasses were sterilized by heating at 140 °C for two hours. A cell suspension of a specified density was applied to the prepared surfaces and placed in the wells of a six-well plate. To ensure reliable cell adhesion, the plate was kept in a CO_2_ incubator for 30 min at 37 °C and 5% CO_2_ concentration. After the adhesion step, fresh nutrient medium was added to each well along with the test composite material samples. The cultivation process continued for 72 h under strictly controlled conditions. Upon completion of the incubation period, the samples were removed, and a detailed microscopic analysis of the state of the cell cultures was performed. For a comprehensive assessment of cell viability, multicolor fluorescence microscopy was employed using a set of dyes: Hoechst 33,342 for nuclear visualization, Rhodamin-123 for visualization of intracellular content (mitochondria), and propidium iodide (PI) for identification of dead cells. Microscopy and documentation of results were performed using a specialized DMI 4000B imaging system (Leica, Wetzlar, Germany), allowing for a comprehensive characterization of the influence of the test samples on the viability and functional activity of the cell culture. Additional details have been described previously [37].

### 2.9. Statistical Processing and Visualization of Experimental Data

The experimental data obtained during the study were subjected to statistical processing using the specialized software package GraphPad Prism ver.8.3.0. Analysis of microphotographs obtained from cell culture studies was performed using ImageJ ver. 1.54f (Fiji) (National Institute of Mental Health) software. All results presented in the work are expressed as mean values with the standard error of the mean, providing a clear display of data variability. To ensure reliability and reproducibility, all conclusions of the study are based on data obtained from at least three independent experiments.

## 3. Results

**Figure 1:**
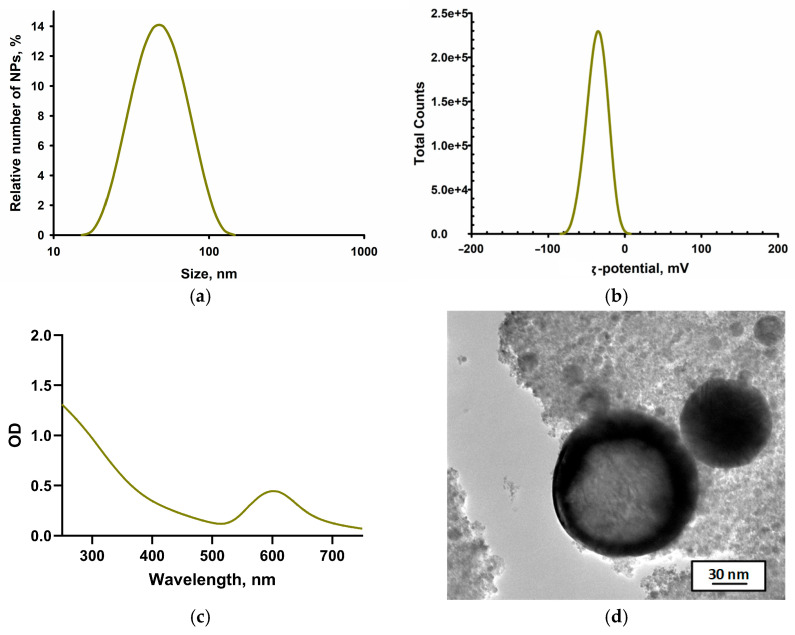
Characterization of the physicochemical properties of Cu NPs obtained by laser ablation. (**a**) Size distribution of nanoparticles obtained by dynamic light scattering. (**b**) ζ-potential distribution of Cu NPs in the colloid. (**c**) Absorption spectrum of the colloid in the UV-Vis range. (**d**) Transmission electron microscopy image of Cu NPs.

It should provide a concise and precise description of the experimental results, their interpretation, as well as the experimental conclusions that can be drawn. Cu NPs were synthesized via pulsed laser ablation. The size of the resulting nanoparticles was confirmed using a Zetasizer Ultra (Malvern Panalytical Ltd., Malvern, UK) particle size analyzer based on the dynamic light scattering (DLS) method. The obtained size distribution was monomodal, with a peak particle size of 47 nm. The distribution exhibited a full width at half maximum of 54 nm (Figure 1a). The electrokinetic potential (ζ-potential) of the synthesized Cu NPs was measured to assess the stability of the colloidal system. The peak of the ζ-potential distribution was determined to be −33 mV (Figure 1b). The optical properties of the Cu NPs colloid were investigated using differential double-beam spectrometry. A broad plasmon resonance band was observed in the visible spectral range, peaking at around 600 nm (Figure 1c). TEM micrographs of the nanoparticles produced by ablation are presented in Figure 1d. The nanoparticles exhibited a spherical-like morphology with well-defined edges. The particle size varied within the range of 20–90 nm.

**Figure 2:**
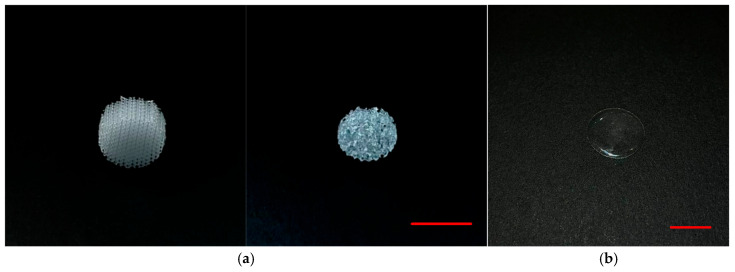
Fabricated specimens from photopolymer resin modified with Cu NPs. (**a**) Three-dimensional samples with lattice and sponge-like architectures. (**b**) A plate-shaped specimen with a diameter of 16 mm. The scale bar in the lower right corner of the photographs corresponds to 10 mm.

The freshly prepared Cu NPs colloid in acetone was introduced into the methacrylate resin at a concentration of 0.1 wt.%. Resins containing 0.01 and 0.001 wt.% Cu NPs were subsequently obtained through serial tenfold dilutions with the pure resin. Various three-dimensional specimens were fabricated from the resulting modified resins using MSLA, as shown in Figure 2. These specimens demonstrated good suitability for mechanical post-processing, including grinding and polishing, and exhibited a high degree of optical transparency. Microscopic analysis confirmed the preservation of facet continuity throughout the entire volume of the constructs and a consistent thickness of approximately 50 µm, even for the composite with the highest Cu NPs content.

The plate specimens were ground and polished to a mirror finish, achieving a distortion-free reflection of objects. Microscopic analysis confirmed that even with the incorporation of the highest investigated concentration of Cu NPs (0.1%), the polished surfaces were free from defects such as cracks, cavities, and craters.

**Figure 3:**
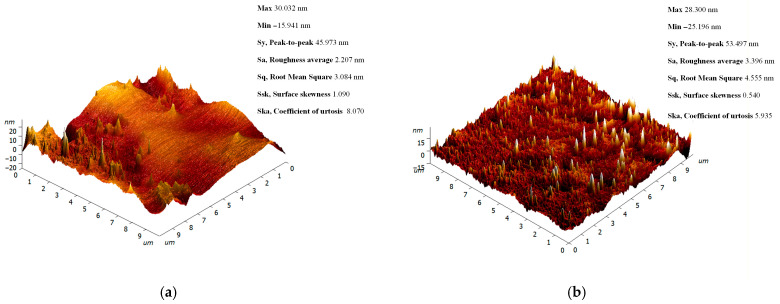
AFM images of 10 × 10 μm surface areas on polished plates fabricated from the unmodified photopolymer resin (**a**) and the modified photopolymer resin containing 0.1% Cu NPs (**b**).

The surfaces of the polished specimens were further examined using AFM. Characteristic three-dimensional surface profiles of the polymer with and without Cu NPs additions are presented in Figure 3. Despite the observed minor changes in quantitative roughness parameters, such as a shift in the height range (Sy) within 45.9–53.5 nm and variations in the average roughness (Sa) within 2.2–3.4 nm, the overall surface character remained unchanged. Key parameters, including the root-mean-square roughness (Sq) and the skewness (Ssk) and kurtosis (Sku) coefficients, exhibit comparable values, indicating the preservation of a similar roughness distribution pattern.

**Figure 4:**
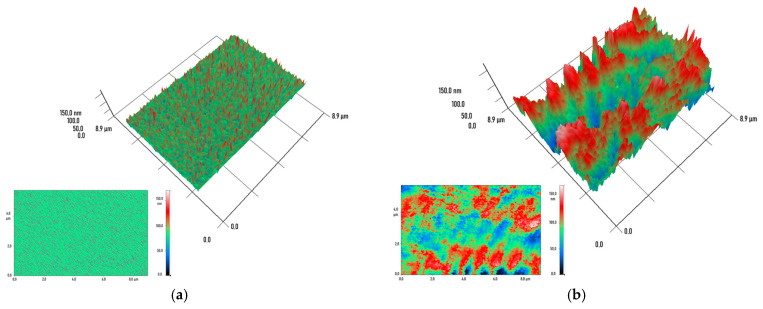

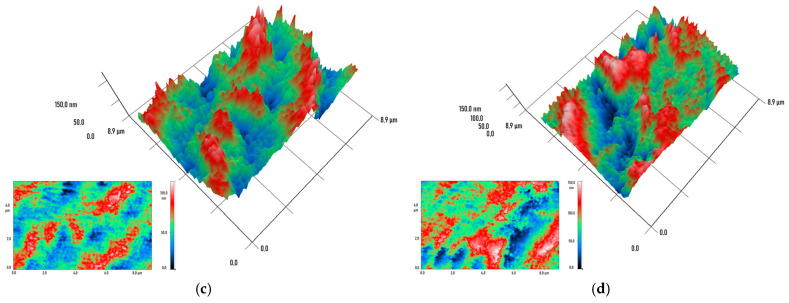
MIM reconstructions of material sample sections fabricated from unmodified photopolymer resin (**a**), and from modified photopolymer resins with Cu NPs concentrations of 0.001% (**b**), 0.01% (**c**), and 0.1% (**d**). The main panels show 3D reconstructions of material cross-sections with dimensions of 8.9 × 8.9 µm, while the original data on which they are based are displayed in the bottom-left insets. The color scale represents the phase difference in transmitted radiation at a wavelength of 405 nm (red—maximum value; blue—minimum value).

The polished specimen surfaces exhibited no significant defects and a high degree of uniformity, as confirmed by atomic force microscopy. However, this technique cannot provide information about internal defects or the degree of Cu NPs dispersion within the printed constructs. Modulation interference microscopy (MIM) was used for contactless and non-destructive study of these volumetric parameters. This method, also known as laser phase microscopy, is based on recording changes in the phase of a light wave transmitted through a transparent or translucent object [38]. These phase shifts are directly related to local variations in the optical density and thickness of the material, allowing for the visualization of internal structural inhomogeneities down to the nanometer level. The method consists of splitting the laser beam into two coherent beams: a reference beam and an object beam [39]. The object beam passes through the sample under study, where its phase is modulated by internal inhomogeneities. It then interferes with the unmodified reference beam, forming an interferogram. A specialized phase modulation device and subsequent computer data processing allow for the reconstruction of a quantitative map of phase shifts, which represents the internal relief of the material’s optical density. In the context of this study, MIM proved to be an indispensable tool for analyzing the internal morphology of the composites. Control samples made from unmodified resin exhibited a disordered, statistical spatial distribution of small-scale phase shifts, typical of an amorphous polymer. However, no distinct regions with a uniform and significant phase shift were observed (Figure 4a). In composite samples containing Cu nanoparticles, MIM clearly revealed the formation of ordered regions with a contrasting phase shift. These regions are interpreted as clusters or zones of local agglomeration of nanoparticles, whose optical density differs significantly from that of the polymer matrix. With increasing filler concentration, a consistent increase in the size of these phase inhomogeneities was observed. Thus, at concentrations of 0.001%, 0.01%, and 0.1%, the average sizes of the detected formations were approximately ~1.0 × 0.5 μm (Figure 4b), ~2.0 × 1.0 μm (Figure 4c), and ~3.0 × 1.0 μm, respectively (Figure 4d).

**Figure 5:**
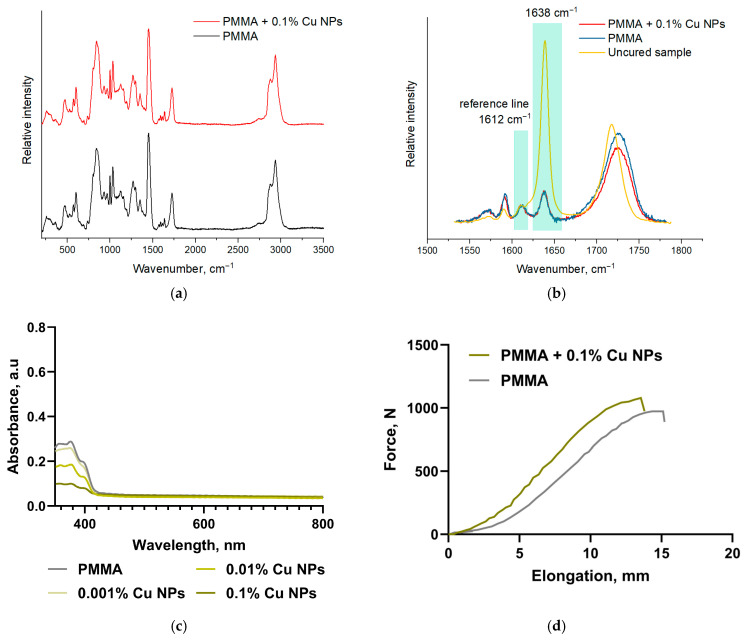
Investigation of the physical properties of specimens fabricated from photopolymer resin with Cu NPs additives. (**a**) Raman spectra of cured resin samples without and with Cu nanoparticles in the 200–3500 cm^−1^ range. (**b**) Raman spectra of cured resin samples without and with Cu nanoparticles, and the uncured original sample in the 1530–1775 cm^−1^ range. (**c**) UV/Vis absorption spectra of composite material specimens with varying Cu NPs content. (**d**) Stress–strain diagrams for samples obtained from pure photopolymerizable resin and modified photopolymer resin containing 0.1% Cu NPs (tensile test).

Figure 5a shows the Raman spectra of products with and without copper nanoparticles at a concentration of 0.1%. Figure 5b shows the Raman spectra of products with and without copper nanoparticles at a concentration of 0.1% over a narrower spectral range. Figure 5b also shows the Raman spectrum of uncured resin. It was found that the spectra of products with and without nanoparticles are quite similar. This indicates that the nanoparticles have no effect on the chemical structure of the cured resin or the formation of additional bonds as a result of their presence.

Using differential double-beam UV-Vis spectroscopy (Figure 5c), it was demonstrated that the addition of Cu NPs did not significantly alter the overall shape of the base polymer’s absorption spectra. However, the intensity of the characteristic peaks in the 380 nm and 400 nm regions decreased with increasing concentration of Cu NPs in the photopolymerizable resin.

A comparative analysis of the tensile stress–strain curves of samples printed from pure photopolymer resin and those modified with 0.1% Cu NPs demonstrated enhanced strength characteristics with modification by nanoparticles, as well as a qualitative change in the deformation and failure patterns of the resulting materials. The initial pure photopolymer exhibits a curve characteristic of a relatively plastic material with a pronounced stage of plastic deformation development: after an initial elastic region and a slight deviation from linearity, the force continues to increase monotonically with increasing elongation, reaching saturation and a plateau around 970 N. In contrast, samples obtained from photopolymer resin modified with 0.1% Cu NPs exhibited a higher peak force value of approximately 1080 N; however, these samples demonstrated reduced plasticity and their failure was more brittle (Figure 5d).

**Figure 6:**
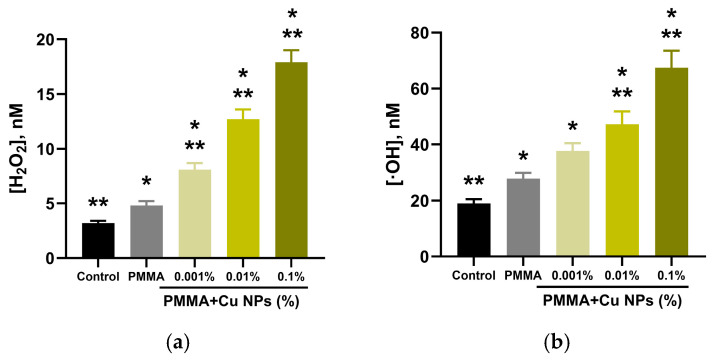
Results of the quantitative analysis of hydrogen peroxide (**a**) and hydroxyl radical (**b**) concentrations formed in aqueous solutions after incubation with polymer material samples containing different weight fractions of Cu NPs. The data are presented as mean ± standard error of the mean from three independent replicates (n = 3). *—significant difference from the control (no polymer), *p* < 0.05; **—significant difference relative to polymer samples containing 0 wt.% Cu NPs, *p* < 0.05.

The influence of plates fabricated from resins modified with Cu NPs on hydrogen peroxide generation was investigated using a highly sensitive enhanced chemiluminescence method (Figure 6a). Under control conditions (in the absence of any material), the hydrogen peroxide concentration was approximately 3 nM. The presence of a sample made from pure, unmodified photopolymer resin in the incubation medium led to a moderate increase in this level to about 5 nM. The incorporation of Cu NPs into the resin composition initiated a statistically significant increase in hydrogen peroxide generation. At the minimum investigated Cu NPs concentration of 0.001%, the amount of detected hydrogen peroxide reached 8 nM. A subsequent increase in the nanoparticles mass fraction to 0.01% and 0.1% was accompanied by a further dose-dependent enhancement of the effect, resulting in the formation of approximately 12 nM and 17 nM of hydrogen peroxide, respectively. These results demonstrate a clear dependence between the nanoparticles concentration in the material and the amount of hydrogen peroxide produced.

To quantitatively assess the generation of hydroxyl radicals upon exposure to the test materials, an investigation was conducted using a highly sensitive fluorescent probe based on coumarin-3-carboxylic acid (Figure 6b). Under control conditions (without samples), the background concentration of hydroxyl radicals was approximately 19 nM. The presence of a sample made from the unmodified composite photopolymer resin led to an increase in the hydroxyl radical concentration to 27 nM. The introduction of Cu NPs into the resin at a concentration of 0.001% promoted a further increase in radical concentration to 38 nM. Statistical analysis indicated that at this Cu NPs concentration, the level of hydroxyl radical generation was not statistically different from the value in the PMMA group, suggesting a possible threshold character for the manifestation of this effect. The obtained data show a clear correlation between the concentration of Cu NPs in the material and the level of highly reactive hydroxyl radical formation.

**Figure 7:**
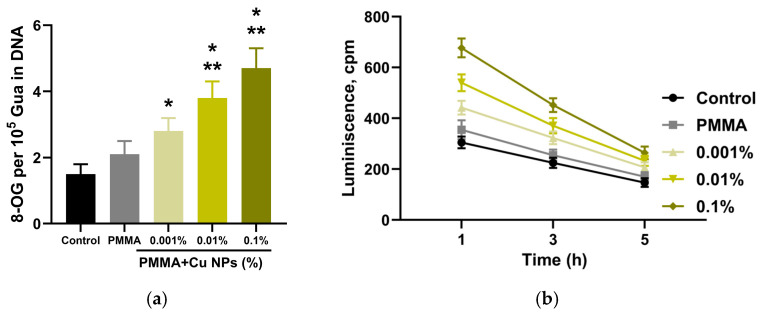
Evaluation of the effects of the synthesized composite materials on biomolecules in vitro. (**a**) Effect of material plate specimens with different Cu NPs content on the concentration of 8-oxoguanine in DNA. (**b**) Level of long-lived reactive protein species (LRPS). Data are presented as mean ± SEM (n = 3). *—significant difference from the control (no polymer), *p* < 0.05; **—significant difference relative to polymer samples containing 0 wt.% Cu NPs, *p* < 0.05.

The influence of material samples, with and without Cu NPs, on the formation of 8-oxoguanine in DNA in vitro was investigated (Figure 7a). Under control conditions (without any polymeric material), the formation of 8-oxoguanine was observed at a level of 1.8 molecules per 10^5^ guanine bases in DNA. The presence of a polymer plate sample not modified with Cu NPs in the incubation medium led to a slight increase in this indicator to approximately 2.4 molecules per 10^5^ guanines in DNA.

The introduction of Cu NPs into the photopolymer resin at a concentration of 0.001% caused an increase in the number of oxidized bases to 2.5 per 10^5^ guanines in DNA, with this value already showing a statistically significant difference from the control group. A subsequent increase in Cu NPs concentration to 0.01% and 0.1% was accompanied by a substantial rise in oxidative DNA damage, expressed by the accumulation of 3.8 and 4.7 molecules of 8-oxoguanine per 10^5^ guanine bases, respectively. The obtained results demonstrate a dose-dependent increase in the level of oxidative DNA modification in the presence of samples containing Cu NPs.

The influence of samples fabricated from photopolymer resin with nanoparticles on the generation of long-lived reactive protein species (LRPS) was studied using the method of induced luminescence. The data presented in Figure 7b show that under control conditions, a low level of LRPS formation is observed. The presence of a polymer matrix sample not containing Cu NPs in the incubation medium did not exert a statistically significant effect on the luminescence level compared to the control.

The incorporation of Cu NPs into the polymer initiated a pronounced dose-dependent effect. The addition of a sample with the minimum filler concentration (0.001%) led to a 45% increase in luminescence intensity relative to the control level. A subsequent increase in the weight fraction of Cu NPs in the photopolymer resin to 0.01% and 0.1% was accompanied by a further enhancement of LRPS generation, exceeding control values by approximately 75% and 2.2-fold, respectively. It is noteworthy that, despite significant differences in the quantity of reactive species formed, their kinetics remained uniform across all investigated groups. The calculated average half-life of LRPS was approximately 5 h and showed no statistically significant variation between groups.

**Figure 8:**
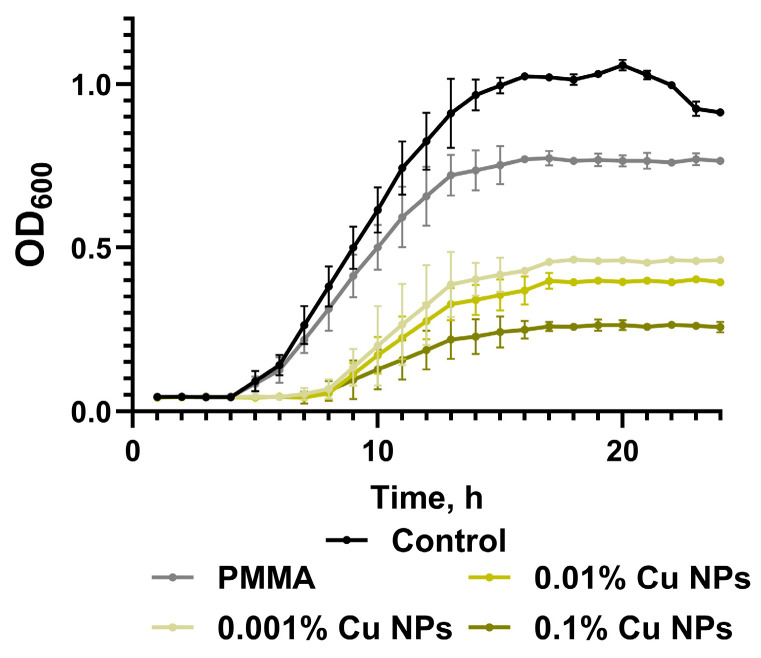
Growth kinetics of *E. coli* suspension cultures cultivated in the presence of the tested polymeric materials with varying nanoparticles content. Data are presented as mean values ± standard error of the mean (n = 3).

A comparative study was conducted to evaluate the effect of composite materials based on photopolymer resins containing different concentrations of Cu NPs on the growth kinetics of *Escherichia coli* suspension cultures (Figure 8). Analysis of the obtained kinetic curves revealed substantial alterations in bacterial growth dynamics depending on the material composition. In the control group, the growth kinetics were characterized by a sequential phase transition: a lag phase of approximately 4 h was followed by a distinct logarithmic phase observed until the 16th hour of cultivation, after which the culture entered the death phase starting at the 20th hour, accompanied by a characteristic decrease in suspension optical density. The presence of samples made from unmodified photopolymer resin in the culture medium did not alter the overall configuration of the kinetic curve compared to the control, but resulted in the absence of a pronounced death phase. In this experimental group, a stationary phase was established from the 15th hour of cultivation, with the final bacterial cell concentration being 15% lower than the control values.

Incubation with samples containing Cu NPs les at a concentration of 0.001% induced a prolongation of the lag phase to 6 h, while the duration of the logarithmic phase remained similar to the control. Despite the general shape of the kinetic curve matching the control group, the final bacterial cell concentration by the 20th hour of cultivation was 55% lower than the control level. A further increase in nanoparticles concentration to 0.01% was accompanied by an extension of the lag phase to 7 h, maintaining its distinctness, while the transition from the exponential to the stationary phase was noted at the 17th hour. The final bacterial cell concentration in the stationary phase was 60% lower than the control level. The most pronounced bacteriostatic effect was observed in the presence of samples with the maximum nanoparticles concentration (0.1%). In this group, the lag phase also lasted 7 h, and the stationary phase was established from the 16th hour of cultivation. A characteristic feature of the kinetic curve in this group was a change in its slope, reflecting a significant slowdown in the increase in suspension optical density. The bacterial cell concentration by the 20th hour of cultivation in this experimental group was 75% lower compared to the control, indicating a pronounced dose-dependent inhibitory effect of Cu NPs on bacterial culture growth.

**Figure 9:**
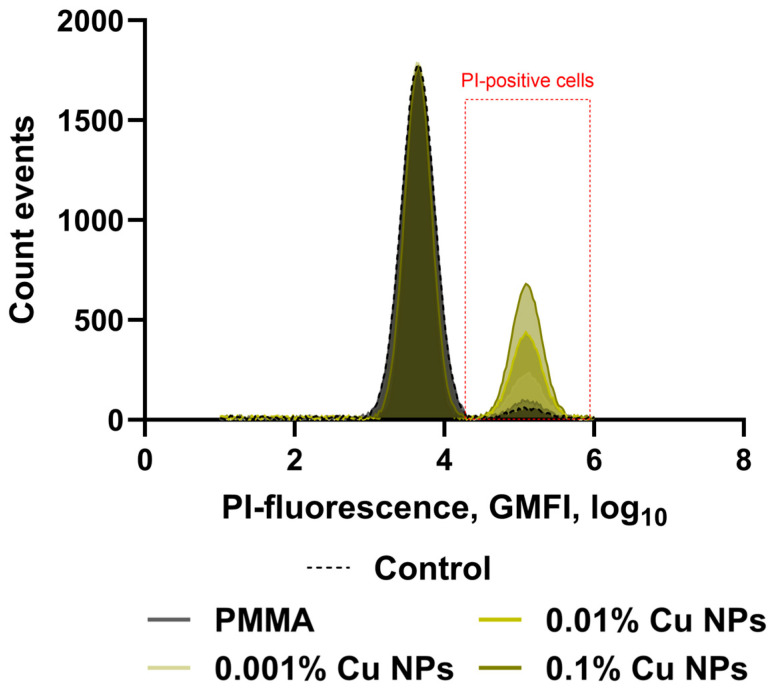
Distribution of bacterial cells based on the geometric mean intensity of propidium iodide fluorescence recorded after incubation with the test material samples. The analytical gate corresponding to the value range characteristic of the PI-positive cell population is highlighted in red.

Analysis of the growth kinetics revealed a substantial decrease in the proliferation rate of bacterial suspension cultures when cultivated in the presence of specimens fabricated from both the unmodified photopolymer resin and the resin modified with Cu NPs. For a more in-depth and differential assessment of the antibacterial activity of the synthesized materials, flow cytometry was employed using the intercalating dye propidium iodide, which selectively penetrates the damaged membranes of non-viable cells (Figure 9). Cytometric analysis demonstrated that in all investigated groups, including the control, as well as samples of the pure polymer and composites with Cu NPs content ranging from 0.001% to 0.1%, the bacterial populations contained both viable and non-viable cells. A statistically significant increase in the proportion of the latter was observed only in the experiment with the sample containing the highest nanoparticles concentration (0.1%).

**Figure 10:**
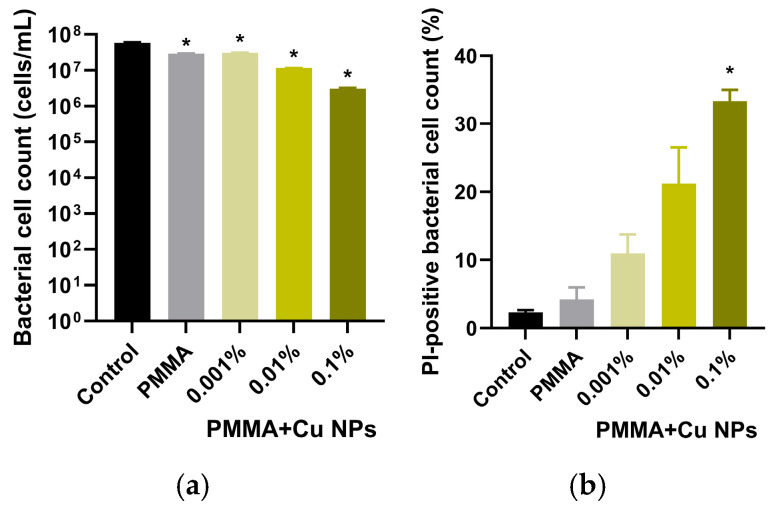
Averaged results obtained by flow cytometry after cultivation of *E. coli* suspension cultures with the test samples. Panel (**a**) shows data on the total number of bacterial cells in the suspension. Panel (**b**) presents the results of the quantitative assessment of PI-positive events. Data are presented as mean ± SEM (n = 3). *—significant difference from the control, *p* < 0.05.

Based on the analysis of histograms showing the distribution of bacterial cells by propidium iodide fluorescence intensity after incubation with samples of unmodified and Cu NPs-modified photopolymer resins, a quantitative determination of both the total bacterial concentration and the proportion of non-viable *E. coli* cells was performed (Figure 10a,b). The obtained data demonstrate a pronounced dose-dependent influence of nanoparticles content on bacterial culture viability. Under control conditions (without samples in the culture medium), the concentration of bacterial cells reached approximately 6 × 10^7^ cells/mL (Figure 10a). Incubation with samples of the unmodified polymer led to a decrease in this value to about 3 × 10^7^ cells/mL, indicating a certain bacteriostatic effect of the base resin.

The use of composite materials with Cu NPs concentrations of 0.001% and 0.01% resulted in further suppression of bacterial growth, with cell concentrations not exceeding values on the order of 10^7^ cells/mL. The most significant antibacterial effect was recorded during incubation with the sample containing 0.1% Cu NPs, where the total cell count was substantially lower. The proportion of PI-positive (dead) bacterial cells increased proportionally with the rising concentration of Cu NPs, reaching approximately 30% when cultured with materials made from the photopolymer resin containing 0.1% Cu NPs (Figure 10b).

A comprehensive assessment of the potential cytotoxic effects of the synthesized materials was conducted using fluorescence microscopy with differential staining of cell cultures. A combined set of fluorescent probes was employed for this purpose: Hoechst 33,342 for nuclear visualization, Rhodamine 123 for labeling intracellular content, and PI for detecting cells with compromised membrane integrity. This combination allows for a reliable evaluation of the cells’ vital status.

**Figure 11:**
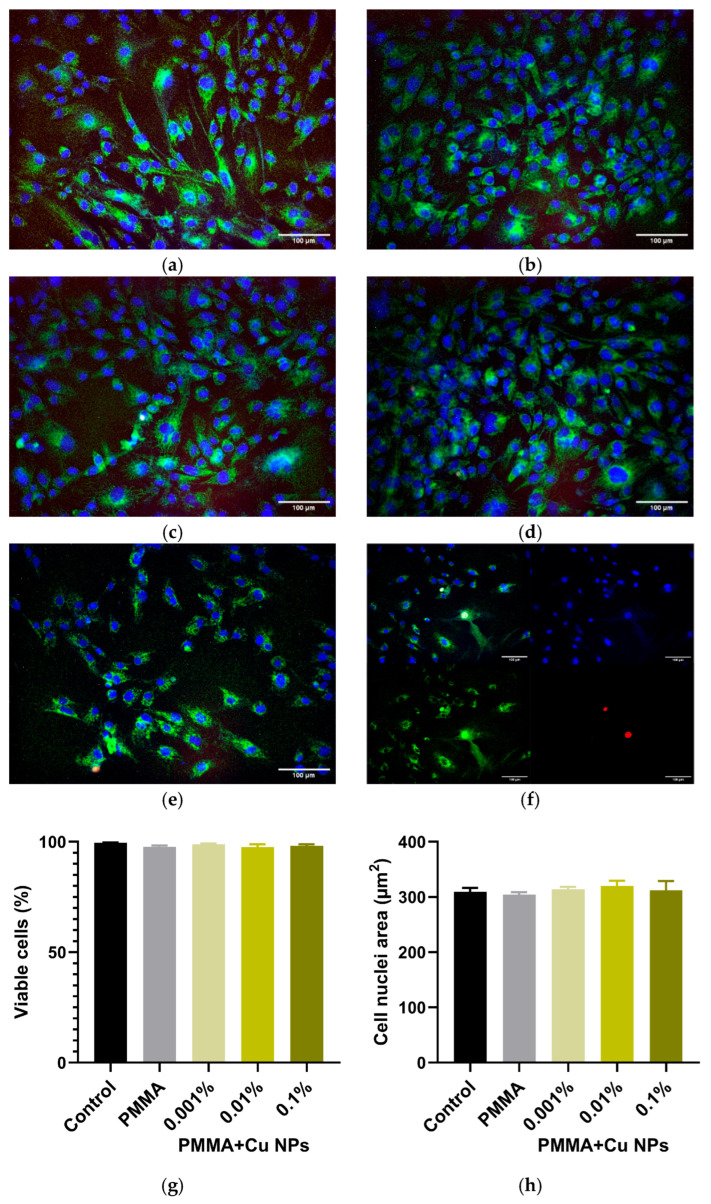
Effect of plates fabricated from photopolymer resin with or without Cu NPs on the growth and development of HSF cells in vitro. (**a**–**e**) Representative micrographs of HSF cell cultures after 72 h (DIV 3) of in vitro cultivation, presented as merged fluorescence images of Hoechst (blue), Rhodamin 123 (green), and PI (yellow): (**a**) cell culture growing on a coverslip (control); (**b**) in the presence of a sample without Cu NPs; (**c**–**e**) cultures growing in the presence of material samples fabricated from modified photopolymer resins with different Cu NPs weight fractions: 0.001% (**c**), 0.01% (**d**), and 0.1% (**e**). (**f**) Visualization of images obtained in three fluorescence channels and their merging. The scale bar in the lower right corner corresponds to 100 µm. (**g**,**h**) Proportion of viable cells (**g**) and nuclear area (**h**) in cultures after 72 h of cultivation in the presence of the tested polymeric materials with varying nanoparticles content. Data are presented as mean ± SEM (n = 3).

Representative micrographs of the cultures on day 3 of in vitro cultivation (DIV 3), taken from the area of direct contact with the material samples, are presented in Figure 11a–f. Qualitative analysis of the series of micrographs did not reveal morphological signs of a cytotoxic effect in any of the experimental groups. The cells maintained their characteristic morphology for this cell type, showing no signs of rounding, process retraction, or pronounced cytoplasmic vacuolization, indicating the absence of acute toxic effects. Quantitative assessment of the proportion of viable (PI-negative) cells confirmed these observations, showing no statistically significant differences between the groups. Even in the presence of the sample with the highest Cu NPs concentration (0.1%), the proportion of viable cells was approximately 97%, only 2% lower than the value in the control group (Figure 11g). Additional morphometric analysis aimed at assessing the cell area also revealed no statistically significant differences between the groups. The mean values of the cell body areas were within a narrow range of 300–320 µm^2^, indicating the preservation of normal morphology and the absence of signs of osmotic cell stress (Figure 11h).

## 4. Discussion

The use of metal and metal oxide nanoparticles is a promising strategy for developing functional materials with enhanced properties [40]. Cu NPs are garnering increasing attention due to their unique combination of catalytic [41] and biological [42] activity.

Our comprehensive analysis of the synthesized Cu NPs indicated a monomodal size distribution with a peak at 47 nm and a distribution width of 54 nm (Figure 1a). The high zeta potential value (−33 mV), characteristic of stable colloidal systems, explains the absence of nanoparticles aggregation in the acetone medium (Figure 1b). The positive surface charge of the particles may be attributed to the adsorption of polar solvent molecules [43]. The optical absorption spectrum (Figure 1c) aligns with theoretical predictions for Cu NPs. According to TEM microscopy data, the colloid predominantly contained spherical particles with well-defined boundaries and sizes ranging from 20 to 90 nm (Figure 1d).

To obtain the modified resins, the freshly synthesized Cu NPs colloid was incorporated into the base photopolymer resin. Using MSLA printing, samples of geometrically complex objects with porous and sponge-like structures (Figure 2a), as well as thin plates with a diameter of 16 mm for comprehensive studies (Figure 2b), were fabricated. The surfaces of plates printed from the photopolymer resin, even with the maximum Cu NPs concentration of 0.1 wt.%, demonstrated optical transparency after polishing, indicating good processability of the material. The obtained samples were free from visible defects, often arising during additive manufacturing [44]. The introduction of Cu NPs within the investigated concentration range did not significantly affect the accuracy of additive manufacturing, did not hinder post-processing, and allowed for the production of objects with complex architecture, which is often challenging for multicomponent polymer systems with nanofillers [45].

AFM was used to analyze potential surface defects on the obtained plates that are not visible to the naked eye. The surfaces of plates printed from resin containing 0.1% Cu NPs exhibited a similar roughness level compared to the control sample printed from resin without Cu NPs (Figure 3a,b). The surfaces lacked pronounced fractures and cracks, showing depressions and protrusions on the order of 20 nm (maximum height variation of 50 nm) (Figure 3b). The AFM data confirm the correct execution of the technological stages of additive manufacturing, grinding, and polishing for the modified resins. However, AFM does not allow for judgment on the spatial distribution of the nanofiller within the material volume or visualization of potential hidden internal defects.

MIM can detect optical inhomogeneities, such as air bubbles, within polymer objects [46], and can also study the distribution of Cu NPs in the sample volume due to the significant difference in the refractive index between the used resin (1.5) and Cu NPs (1.3) [47]. While samples of plates without Cu NPs additions showed an absence of any optical inhomogeneities (Figure 4a), samples obtained from resins with added Cu NPs exhibited phase inhomogeneities, the sizes of which depended on the Cu NPs concentration (Figure 4b–d). This result indicates probable nanoparticles aggregation processes within the matrix. The progressive increase in the average size of inhomogeneities from ~1.0 × 0.5 µm to 3.0 × 1.0 µm with increasing Cu NPs concentration from 0.001% to 0.1% suggests that during photopolymerization, a small fraction of Cu NPs may form ordered structures. Of note, the spatial distribution and aggregation state of Cu NPs within the polymer matrix exert a complex influence not only on their stability but also directly determine their reactivity and propensity to release active ions into the environment, particularly under exposure to atmospheric moisture and oxygen [48]. Experimental data obtained from long-term studies confirm this relationship. For instance, incubation of polypropylene composites containing 5 wt.% nanoparticles in deionized water revealed a two-fold increase in the concentration of leached copper ions by day 10 compared to their level after the first 24 h, indicating a progressive nature of the migration process [49]. A similar trend was observed in the polyethylene/Cu NPs (5.0 wt.%) system, where the quantitative ion release amounted to 18 mg/L·cm^2^ and 26 mg/L·cm^2^ on days 1 and 10, respectively, suggesting ongoing corrosion of the nanoparticles within the polymer bulk [50]. An important aspect is the behavior of such nanocomposites upon contact with real media. Studies on composite films based on low-density polyethylene (LDPE) demonstrated that the release of copper ions into aqueous and alcoholic food simulants (10% ethanol) was statistically insignificant. However, a key finding of this work was the absence of chemical bonding between the surface of the Cu NPs and the LDPE polymer matrix [51]. This implies that the stabilization of the particles in this case is purely physical and kinetic in nature, governed by steric hindrances and limited diffusion, which could potentially lead to migration under altered service conditions, for example, in acidic environments.

A common issue for resins used in additive manufacturing is the presence of residual monomer content after photopolymerization [52,53,54]. This problem is particularly relevant for the additive manufacturing of medical devices and materials in direct contact with living tissues, due to the pronounced toxic effects of acrylate monomers [55,56]. The primary mechanism of toxicity is associated with the high electrophilicity of acrylate monomers, which readily react with nucleophilic groups of biological macromolecules, including thiol groups in proteins and amino groups in nucleic acid bases [57,58]. This can lead to the induction of oxidative stress, disruption of cell membrane functions, and initiation of apoptosis. For monomers like methyl methacrylate, the ability to cause contact dermatitis and respiratory irritation has been demonstrated, while some oligofunctional acrylates exhibit mutagenic properties in vitro [59,60].

Figure 5a,b shows the Raman spectra of resin products without nanoparticles, with Cu nanoparticles, and the uncured original sample. The line in the Raman spectra of the samples with a frequency of 1612 cm^−1^ refers to the stretching vibrations of C=C bonds in aromatic groups. The number of bonds in such groups does not change during polymerization. In view of this, the line with a frequency of 1612 cm^−1^ is considered an internal standard for assessing the degree of conversion. The Raman line with a frequency of 1638 cm^−1^ corresponds to the stretching vibrations of aliphatic C=C bonds in acrylic groups [61]. The spectra were normalized to the line 1612 cm^−1^. The degree of conversion of C=C bonds in the samples was determined from the Raman spectra using the formula:

$$DC=\frac{I_{o(1638)}/I_{o(1612)}}{I_{r(1638)}/I_{r(1612)}}$$

where *I*_o(1638)_, *I*_o(1612)_ are the integrated intensities of the 1638 and 1612 cm^−1^ lines of the cured sample (PMMA and PMMA + 0.1% Cu NPs), *I*_r(1638)_, *I*_r(1612)_ are the integrated intensities of the 1638 and 1612 cm^−1^ lines of the uncured sample. Spectral line decomposition was performed according to the procedure described in detail in [62]. For both samples (PMMA and PMMA + 0.1% Cu NPs), the degree of conversion of C=C bonds is approximately 94 ± 1%.

The absence of significant changes in the spectral characteristics of the materials in the UV and visible ranges likely indicates the preservation of the main optical properties and chromophoric characteristics of the obtained samples (Figure 5c).

Mechanical tests demonstrated an unsignificant increase in the strength properties of the polymer matrix with the addition of 0.1% Cu NPs and a decrease in ductility (Figure 5d). The change in mechanical properties can be influenced by multiple factors: printing-induced anisotropy, thermal properties changing, the intrinsic reinforcing and/or plasticizing effects of nanoparticles [63,64,65]. The literature data also indicate an increased efficiency of monomer conversion of photopolymer resins after addition of NP metals. The observed effect may be attributed to several simultaneous factors: activation of photoinitiators by Cu NPs via electron or excitation energy transfer, leading to an increased concentration of active radical centers in the system [66]; influence of nanoparticles on the rheological properties of the reaction medium during polymerization due to altered segmental mobility of growing polymer chains [67]; and localized enhancement of photoinitiator molecule activation in close proximity to the nanoparticles [68].

Since copper is a transition metal, its nanoparticles can act as photosensitizers and catalysts for redox reactions, participating in the generation of ROS via Fenton-like reactions [69,70]. This work quantitatively assessed the formation of two types of ROS: hydrogen peroxide—the most stable form of ROS (Figure 6a)—and hydroxyl radicals—the most reactive form (Figure 6b). In aqueous environments with the addition of samples containing no Cu NPs, a slight increase in the concentration of the studied ROS was observed. Incubation of media with samples containing Cu NPs showed a pronounced, dose-dependent increase in the measured ROS levels proportional to the weight fraction of the nanoparticles.

It is noteworthy that ROS are formed under the influence of various physical factors, including ionizing [71] and UV [72] radiation, ultrasound [73], low-temperature plasma [74], various electromagnetic waves [75], high temperatures [76], and various mechanical impacts [77], among others. Transition metals can potentially sensitize the effects of the aforementioned physical factors [78]. An increase in ROS levels can, on one hand, stimulate biological systems [79], but on the other hand, if the cell’s antioxidant capacity is exceeded, ROS can lead to significant problems [80]. This work demonstrates (Figure 7) that in the presence of the photopolymer resin, more intense damage to DNA and proteins occurs compared to the control, while Cu NPs exert a weak sensitizing effect.

The obtained results demonstrated a significant inhibitory effect of the materials against planktonic cultures of *E. coli* (Figure 8). Flow cytometry data quantitatively confirm the conclusions drawn from the growth kinetics analysis and show that the incorporation of Cu NPs into the photopolymer resin potentiates its antibacterial properties in a concentration-dependent manner, reaching maximum efficacy at the highest investigated filler concentration (Figure 9 and Figure 10). The antibacterial action of Cu NPs is well-documented in the literature and is attributed to a number of mechanisms, often acting simultaneously [81]. Besides the aforementioned ROS-inducing action of Cu NPs, accompanied by oxidative damage to key groups of biomacromolecules (nucleic acids, proteins, lipids), Cu NPs, due to their high surface charge and significant specific surface area, effectively adsorb onto membranes, disrupting the integrity of the lipid bilayer and increasing its permeability [82,83]. This is accompanied by a loss of cellular homeostasis, uncontrolled leakage of potassium ions and intracellular components, ultimately leading to cell lysis. Another described mechanism is the induction of ionic imbalance due to the release of copper ions (Cu^+^/Cu^2+^) from the nanoparticles, accompanied by disruption of transmembrane transporter function, competitive inhibition of the uptake of essential trace elements (Zn^2+^, Mn^2+^), and inactivation of metal-dependent enzymes [84,85]. Upon penetrating the cytoplasm, copper ions interact with thiol groups of key proteins, causing denaturation and loss of their functional activity [86].

Notably, alongside the antibacterial action of the obtained materials, no significant cytotoxic impact on fibroblast cell cultures was detected (Figure 11): morphological signs of cellular stress were absent, and cell viability indicators, as well as nuclear sizes, did not differ significantly across all groups. According to the literature, Cu NPs are toxic to cell cultures, causing oxidative stress, cytotoxicity, genotoxicity, immunotoxicity, neurotoxicity, and inflammation [87]. It is worth noting that experimental design for assessing the impact of Cu NPs on living systems is methodologically challenging, as highly reactive Cu NPs in biological environments and fluids are instantly oxidized to the more stable form—copper oxide—and rapidly undergo biocoronation—a process where biomolecules adsorb onto the particle surface. The observed good cytocompatibility of the developed composite materials appears, at first glance, contradictory to the established data on Cu NPs toxicity. In particular, Prabhu et al. reported that exposure of dorsal root ganglion (DRG) neuron cultures to Cu NPs resulted in pronounced vacuolization, partial detachment of some neurons from the substrate, and disruption of the neurite network. Furthermore, Cu NPs exposure exerted significant toxic effects for all investigated sizes (40, 60 and 80 nm) compared to untreated control cultures [88]. Similarly, Na and Kennedy demonstrated differential effects of three distinct size fractions of Cu NPs (25, 40–60, and 60–80 nm) on A549, HepG2, and SH-SY5Y cell lines. It was noted that all tested Cu NPs fractions exhibited cytotoxicity towards all three cell lines; however, particles in the 40–60 nm range appeared to exert a stronger effect on cells, potentially due to a smaller hydrodynamic radius in the cell culture medium [89].

One critically important factor determining the cytotoxic properties of Cu NPs is their chemical form and the release environment. Pure Cu NPs in aqueous media are often prone to rapid aggregation and corrosion, which enhances their toxic effect. One reported method to reduce Cu NPs cytotoxicity is their combined use with CuO NPs, which are less cytotoxic and more bioinert. In particular, Rodhe et al. showed that Cu NPs exhibited higher toxicity towards HL60 leukemia cells compared to both CuO NPs and dissolved copper (CuCl_2_), as well as a faster release of copper compared to CuO NPs at a maximum concentration of 100 µg/mL [90]. Cu/CuO nanoparticles suppressed the proliferation and viability of both normal (WI-38) and carcinoma (A549) lung cells. Treatment of both cell types with Cu/CuO nanoparticles at a concentration equal to the IC_50_ (201 µg/mL) for both cell lines suppressed their proliferation and viability [91]. In a composite material, the polymer matrix can stabilize the particles, preventing their aggregation and ensuring predictable surface chemistry. Moreover, the very nature of the polymer (its hydrophilicity or hydrophobicity), the presence of functional groups capable of coordinating with copper ions, and the degree of cross-linking directly affect ion availability. For instance, Palza et al. reported very low cytotoxicity towards normal mouse fetal cortical brain cells and human osteosarcoma (SAOS-2) cells for hydrogel-based materials containing Cu NPs; this effect was observed even at a Cu NPs concentration of 0.5 wt.%, whereas no effect was observed when using polypropylene as the matrix, even at Cu NPs concentrations up to 20 wt.% [23]. In turn, functionalization of Cu NPs with starch contributed to a 1.9-fold reduction in their cytotoxicity towards mouse embryonic fibroblast (3T3L1) cells compared to pure Cu NPs, and a 1.45-fold reduction compared to the ionic form of copper at a maximum concentration of 100 µg/mL [92]. Therefore, the utilization of polymer matrices facilitates controlled release kinetics of both ionic and particulate copper species into the local microenvironment, ensuring predictable cytotoxic responses of the material.

Our results, demonstrating the absence of pronounced cytotoxicity even at high Cu NPs concentrations, may be due to the immobilization of Cu NPs within the polymer matrix, which limits direct interaction with cell membranes and reduces the rate of copper ion release. Furthermore, the observed effect might reflect the compensatory activation of cellular antioxidant defense systems, mitigating the potential pro-oxidant action of the nanoparticles.

Consequently, our materials could potentially serve as a basis for developing a new generation of biomedical materials combining the functional properties of Cu NPs with high biocompatibility. A promising direction is the creation of implantable devices and wound coatings where controlled release of copper ions could provide an antimicrobial effect without compromising the viability of surrounding tissues. However, practical realization of this potential requires further research focused on studying long-term effects and mechanisms of cellular adaptation in vivo.

## 5. Conclusions

This study demonstrates the successful development of a functional photopolymer composite modified with Cu NPs synthesized by laser ablation. The obtained nanoparticles were characterized by stability in colloidal form, spherical morphology, and a narrow size distribution. Their incorporation into the methacrylate resin via MSLA printing enabled the fabrication of complex architectural objects with high-quality surfaces free of visible defects. A key achievement of this work is the revelation of the dual influence of Cu NPs within the polymer matrix. On the one hand, they exhibited pronounced concentration-dependent pro-oxidant and antibacterial activity against *E. coli*. On the other hand, and most importantly, the immobilization of the nanoparticles in the polymer mitigated their cytotoxicity towards human fibroblast cultures, indicating the high biocompatibility of the material. In addition, Cu NPs significantly increase the strength of printed products from the modified resin used. The results obtained in this work demonstrate the fundamental feasibility of additively manufacturing materials based on photopolymerizable acrylate resin with the addition of Cu NPs, where the polymer matrix performs not only a structural but also a regulatory function, controlling the biological activity of the nanoparticles. This opens new horizons for the development of intelligent materials with programmable biological properties.

## Data Availability

The raw data supporting the conclusions of this article will be made available by the authors upon request.
